# The Epstein–Barr virus lytic cycle activator Zta interacts with methylated ZRE in the promoter of host target gene *egr1*

**DOI:** 10.1099/vir.0.007922-0

**Published:** 2009-06

**Authors:** James Heather, Kirsty Flower, Samine Isaac, Alison J. Sinclair

**Affiliations:** School of Life Sciences, University of Sussex, Brighton BN1 9QG, UK

## Abstract

Activation of the host gene *egr1* is essential for the lytic replication of Epstein–Barr virus (EBV). *egr1* is activated by Zta (BZLF1, ZEBRA). Zta interacts directly with DNA through a series of closely related Zta-response elements (ZREs). Here we dissect the mechanism used by Zta to interact with the *egr1* promoter and identify a weak interaction with *egr1*ZRE that is dependent on the distal part of *egr1*ZRE. Furthermore, we demonstrate that the ability of Zta to interact with *egr1*ZRE is enhanced at least tenfold by methylation. The ability of Zta to transactivate a reporter construct driven by the *egr1* promoter can be enhanced by methylation. As the ability of Zta to interact with a methylated ZRE in the EBV genome correlates with its ability to activate the expression of the endogenous viral gene *BRLF1*, this suggests that Zta may also have the capability to overturn epigenetic control of *egr1*.

Following infection, Epstein–Barr virus (EBV) establishes long-lived latency in memory B-lymphocytes (Thorley-Lawson *et al.*, 1996) and other lineages (Chaganti *et al.*, 2008; Conacher *et al.*, 2005). Reactivation from latency can occur from both memory B-lymphocytes and tumour cells, induced by a variety of physiological stimuli (Miller, 1989). The key regulator of EBV lytic replication, Zta (*BZLF1*, Zta, ZEBRA) is a virally encoded transcription and replication factor which interacts directly with DNA through its basic region both in Zta-regulated promoters and within the origin of lytic replication (Feederle *et al.*, 2000; Miller *et al.*, 2007; Sinclair, 2003, 2006; Sinclair & Farrell, 1992; Speck *et al.*, 1997). Zta also interacts indirectly with DNA and has the potential to alter gene expression through its interactions with some host transcription factors (Gutsch *et al.*, 1994; Sista *et al.*, 1993, 1995; Swenson *et al.*, 2001; Wu *et al.*, 2003; Zhang *et al.*, 1994).

Zta interacts with a subset of ZREs that contain CpG motifs (Bhende *et al.*, 2004). Methylation of transcription factor binding sites at CpG motifs is normally either inhibitory for binding (BSAP, ATF, YY1, EPO80) or neutral (SP1, RFX) (Falzon & Kuff, 1991; Holler *et al.*, 1988; Iguchi-Ariga & Schaffner, 1989; Mancini *et al.*, 1998; Tierney *et al.*, 2000). Methylated DNA is generally associated with non-expressed genes and the inability of some transcription factors to interact with methylated DNA may play a role in either silencing, or maintaining the inhibition of, gene expression. In contrast, Zta has the unusual property of displaying enhanced binding to methylated ZREs (Bhende *et al.*, 2004, 2005; Karlsson *et al.*, 2008a, b; Petosa *et al.*, 2006; Wang *et al.*, 2005).

The host gene *egr1* encodes a transcription factor which is induced following EBV lytic cycle activation, through the action of Zta (Chang *et al.*, 2006). *egr1* is involved in diverse biological functions (Sukhatme *et al.*, 1988). Mutational analysis of the promoter suggests that activation by Zta occurs through a serum response element (SRE) that is flanked by two Ets response elements and through a tandem pair of potential ZREs (Fig. 1). Zta activates Erk, a member of the MAP kinase family, resulting in transactivation through the Ets response elements (Chang *et al.*, 2006). Indeed, mutation of either the Ets or ZRE sites impacts upon Zta activation *in vivo* (Chang *et al.*, 2006), suggesting that both direct and indirect mechanisms are used to activate expression through Zta.

The *egr1* promoter contains tandem copies of potential ZREs. A mutation covering both sites was shown to decrease Zta transactivation *in vivo* (Chang *et al.*, 2006); however, the precise sequence elements required for Zta to interact with the *egr1* promoter have not been mapped. In order to investigate the molecular mechanism by which Zta can directly activate *egr1* expression, we questioned the contribution from each ZRE for the interaction with Zta.

A schematic diagram of the *egr1* promoter region is shown in Fig. 1. The ability of Zta to interact with the *egr1*ZRE was assessed using electrophoretic mobility shift assays (EMSA). A Zta–DNA complex was readily detected between Zta and a previously characterized ZRE from the EBV *BRLF1* promoter (RpZRE2) (Fig. 1c[Fig f1]). The presence of a Zta complex with the *egr1*ZRE probe, which spans both ZREs, was difficult to detect in comparison, although long exposures revealed a weak protein–DNA complex. From titration experiments we demonstrate that Zta forms a complex with the *egr1*ZRE probe that is at least 20-fold weaker than that formed with three examples of previously characterized ZREs: RpZRE2, RpZRE1 and AP1 (Fig. 1d[Fig f1]). The individual mutations of the tandem ZRE sequences reveal that both contribute to complex formation (Fig. 1c[Fig f1]); mutation of the proximal site results in a modest reduction in binding, whereas mutation of the distal site ablates Zta complex formation under these conditions.

Intriguingly, the distal *egr1*ZRE contains a CpG motif (Fig. 2a[Fig f2]). Zta has the unusual property of being able to recognize response elements when methylated at CpG motifs; indeed, the interaction between Zta and two previously characterized ZREs from the *BRLF1* promoter, RpZRE2 and RpZRE3, is strongly enhanced by methylation (Bhende *et al.*, 2004, 2005; Karlsson *et al.*, 2008b). *egr1* is methylated in B-cells, yet a combination of demethylation agents and physiological stimuli is able to induce *egr1* expression (Seyfert *et al.*, 1990). This demonstrates that methylation can play a role in the control of *egr1* gene expression.

To assess whether methylation has any effect on the interaction between Zta and the *egr1*ZRE, we generated unmethylated and methylated *egr1*ZRE probes and assessed the interaction of each with Zta by EMSA (Fig. 2). Methylation of the single CpG motif in the distal ZRE clearly enhanced DNA binding and titration experiments revealed that at least tenfold more Zta–DNA complex is formed with the methylated *egr1*ZRE compared with the unmethylated *egr1*ZRE.

Molecular modelling predicts that the methylated cytosines of RpZRE2 and RpZRE3 make contact with Zta amino acid residues S186, C189 and R190 as indicated in Fig. 3 (Bhende *et al.*, 2005; Karlsson *et al.*, 2008a; Petosa *et al.*, 2006). Indeed, S186 and C189 have been shown to aid binding to methylated RpZRE2 and RpZRE3 experimentally (Bhende *et al.*, 2005; Karlsson *et al.*, 2008a, b; Wang *et al.*, 2005). In addition to their relevance for interaction with methylated ZREs, mutations of C189 and S186 promote differential binding to some non-CpG-containing ZREs (summarized by Heston *et al.*, 2006).

The distal *egr1*ZRE, containing the CpG motif, has strong homology with RpZRE2 (6/7 of the heptad core), and significant homology with RpZRE3 (4/7 of the heptad core), suggesting that the same amino acid residues may contribute to interaction with the methylated CpG motif. As only the rightwards CpG motif of RpZRE3 is conserved within the distal *egr1*ZRE, it is relevant to question the contribution of amino acids C189 and S186 to the DNA-binding function. To assess this, we used single point mutants of Zta, ZtaC189A and ZtaS186A, to investigate their impact on the interaction of Zta with methylated *egr1*ZRE. The full-length version of each protein was generated in an *in vitro* translation system (Fig. 3) and the interaction of equivalent amounts of each with methylated *egr1*ZRE probe was analysed by EMSA. Mutation of either S186 or C189 greatly reduced the ability of Zta to interact with the methylated *egr1*ZRE.

To assess whether methylation of the *egr1* promoter affects the ability of Zta to activate it, we co-expressed Zta and a previously characterized *egr1* promoter construct (Chang *et al.*, 2006) in two epithelial cell lines. A comparison was undertaken between methylated and non-methylated versions of the promoter construct (Fig. 3e[Fig f3]). In HeLa cells, Zta did not activate expression of the untreated plasmid, yet Zta activated the methylated reporter construct approximately fourfold. This clearly demonstrates that Zta can preferentially activate the methylated *egr1* promoter construct.

We note that the Zta activation occurs against a background of decreased Zta-induced activity of the *egr1* promoter plasmid that had undergone a ‘mock-methylation’ reaction. We also noticed a reduction in the basal activity of plasmids that have been methylated and a partial reduction in basal activity of those plasmids that have been through the mock-methylation reaction in all cell types tested (data not shown). This mirrors what has been described previously for the *BRLF1* and *BZLF1* promoters in 293T and DG75 cells (Bhende *et al.*, 2004, 2005).

In contrast, in U2OS cells, Zta activation of expression of the *egr1* promoter plasmid was approximately twofold for both the untreated and the methylated plasmids, suggesting no methylation-dependent enhancement (Supplementary Fig. S1, available in JGV Online). This analysis suggests that the enhanced activation by Zta from the methylated *egr1* promoter is cell-line dependent.

Taken together, these data show that Zta is able to interact with the *egr1*ZRE through the distal ZRE and furthermore that methylation of the CpG motif in the distal element does not prevent the interaction with Zta, but rather enhances binding. The methylation sensitivity of the interaction of Zta with *egr1*ZRE and the dependence on S186 and C189 resembles its binding with RpZRE3 (Karlsson *et al.*, 2008a, b). The *BRLF1* promoter is methylated during viral latency (Bhende *et al.*, 2005) and the ability of Zta to interact with methylated RpZRE3 correlates with its ability to activate expression of *BRLF1* in a B-cell line (Karlsson *et al.*, 2008a, b). The demonstration that activation of the *egr1* promoter by Zta can be enhanced by methylation suggests that Zta may be able to activate the *egr1* promoter *in vivo*, when it is methylated. Thus the potential arises that Zta is able to overturn the epigenetic silencing of both a viral promoter (*BRLF1*) and a host promoter (*egr1*).

## Supplementary Material

[Supplementary figure]

## Figures and Tables

**Fig. 1. f1:**
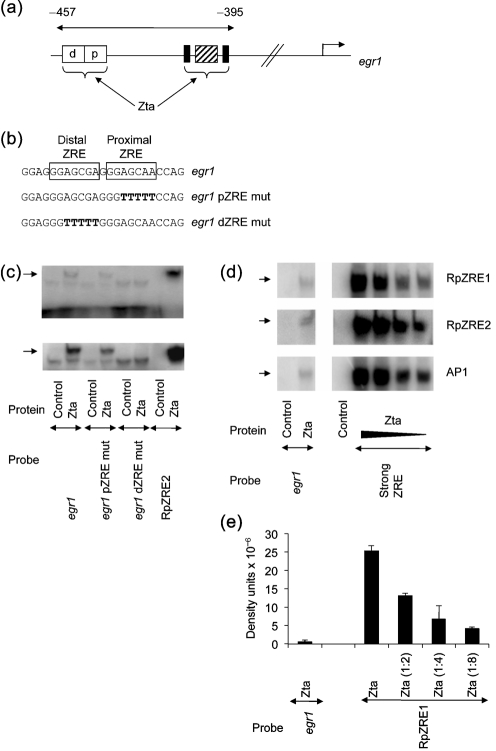
Interaction of Zta with *egr1*ZREs. (a) The organization of the *egr1* promoter is shown schematically. The numbers represent distance from the transcription initiation site. The Ets response elements are shown by filled boxes, with the SRE hatched. The open boxes represent the tandem ZREs, proximal (p) and distal (d). (b) The nucleotide sequence of one strand of the oligonucleotide spanning the ZREs is shown. *egr1* represents the wild-type sequence, *egr1* dZRE mut, the mutation in the distal ZRE and *egr1* pZRE mut, the mutation in the proximal ZRE. Double-strand versions of these sequences were used as probes for EMSA. (c) EMSA analysis of the interaction between Zta and *egr1*, *egr1* pZRE mut, *egr1* dZREmut and RpZRE2 was undertaken as described previously (Schelcher *et al.*, 2005, 2007). Probes were labelled to approximately equivalent specific activities and the relative amounts used in the reactions are: *egr1* (1.0), *egr1* pZRE mut (0.5), *egr1* dZRE mut (0.7) and RpZRE2 (0.3). In these experiments the probe is in excess, as shown by the dose response in (d) and (e). Zta protein was generated in a wheatgerm translation system, and its interaction with a labelled probe was compared with an unprogrammed lysate as control. For each panel, complexes were separated on the same gel by electrophoresis and visualized by phosphoimaging. The exposure of the bottom panel was ten times longer than that shown in the top panel to visualize the weak Zta–*egr1* protein–DNA complexes. The experiment was repeated with similar results. (d) EMSA analysis of the interaction between Zta and *egr1*, compared with known strong ZREs, RpZRE1 (GATCTCTTTTATGAGCCATTGGCA), RpZRE2 (GATCAAGCTTATGAGCGATTTTTAT) (Bhende *et al.*, 2004) and AP1 (GATCCATGACTCAGAGGAAAACATACG) (Hicks *et al.*, 2003; Kouzarides *et al.*, 1991), were undertaken as in (c). The relative amounts of probes used in the reactions are equivalent. The experiment was repeated with similar results. (e) Quantification of the complexes for RpZRE1 and *egr1*. These were detected by phosphoimaging in two experiments. Error bars indicate sd.

**Fig. 2. f2:**
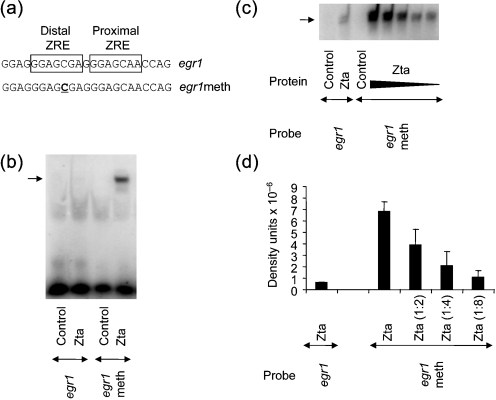
Interaction of Zta with methylated *egr1*ZRE. (a) The nucleotide sequence of one strand of the oligonucleotide spanning the ZREs is shown, together with the methylated version (underlined). Double-strand versions of these sequences were used as probes in EMSA. (b) EMSA analysis of the indicated proteins with the *egr1* probes was carried out as described in Fig. 1. Probes were labelled to approximately equivalent specific activities and the relative amounts used in each comparison are *egr1* (1.0) and *egr1*meth (0.72). The experiment was repeated with similar results. (c) EMSA analysis of the interaction between Zta and *egr1*, compared with the methylated *egr1* was undertaken using serial dilutions of Zta protein. The experiment was repeated with similar results. (d) Quantification of the complexes for *egr1*meth and *egr1* (density units×10^−6^) after adjustment for the relative amounts of probes used for each EMSA. These were detected by phosphoimaging in two experiments. Error bars indicate sd.

**Fig. 3. f3:**
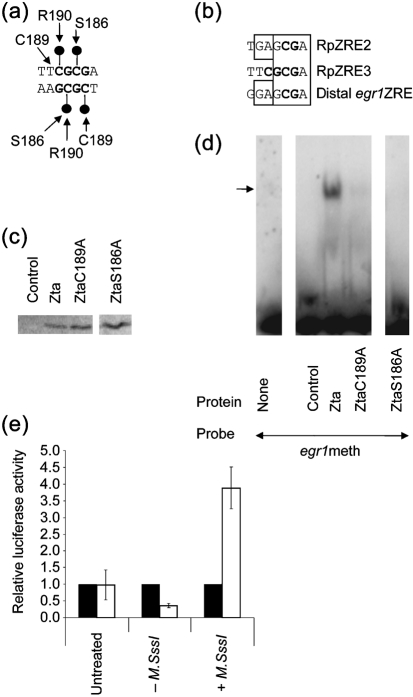
Relevance of serine 186 and cysteine 189 for interaction with methylated *egr1*ZRE and effect of methylation on Zta activation of promoter. (a) Schematic diagram summarizing the known interactions between serine 186, cysteine 189 and arginine 190 with methylated cytosines (indicated as balls) and between cysteine 189, and with the methyl group of a thymidine residue in RpZRE3. (b) Alignment of RpZRE2, RpZRE3 and the distal *egr1*ZRE. Nucleotides conserved between the distal *egr1*ZRE with either RpZRE2 or RpZRE3 are highlighted with boxes. (c) Zta, ZtaC189A and ZtaS186A (Karlsson *et al.*, 2008a) were generated in a wheatgerm translation system, together with trace levels of [^35^S]methionine, and fractionated by SDS-PAGE. The expression levels were determined and equivalent amounts were used for EMSA analysis. (d) The interaction of the indicated Zta proteins, or unprogrammed lysate as control, with the methylated *egr1*ZRE probe was determined by EMSA. The experiment was repeated with similar results. (e) HeLa cells were transfected with the indicated plasmids using Effectene (Qiagen). After 48 h cell extracts were prepared and assayed for luciferase activity using the luciferase assay system (Promega). The data for each type of plasmid [luciferase activity (μg protein)^−1^] are expressed relative to the activity levels seen in the absence of Zta, together with the sd, which was derived from at least two experiments. The reporter plasmid used was *egr1* (−504/+9)LUC (Chang *et al.*, 2006). The plasmid was transfected in an untreated form, or it underwent a mock methylation reaction (−*M.SssI*), or it went through a methylation reaction with the methyl transferase *M.SssI* (New England Biolabs) (+*M.SssI*), both overnight, followed by purification on a QIAprep column (Qiagen). Prior to transfection, the extent of methylation was evaluated by a diagnostic digestion with the methylation-sensitive restriction enzyme *Bst*UI. The open bars represent transfections undertaken with pBabe BZLF1 (Hicks *et al.*, 2003), while the filled bars represent transfections undertaken with the ‘empty’ pBabe vector.
