# Supplementary material for: The Epstein–Barr virus lytic cycle activator Zta interacts with methylated ZRE in the promoter of host target gene egr1
Source: J Gen Virol. 2009 Jun;90(Pt 6):1450–4. doi: 10.1099/vir.0.007922-0 (PMC2885059; doi:10.1099/vir.0.007922-0)
Supplement: [Supplementary figure] [file supp_90_6_1450__index.html]

 The Epstein-Barr virus lytic cycle activator Zta interacts with methylated ZRE in the promoter of host target gene egr1 -- Heather et al. 90 (6): 1450 Data Supplement - Supplementary figure -- Journal of General Virology

### The Epstein–Barr virus lytic cycle activator Zta interacts with methylated ZRE in the promoter of host target gene *egr1*, by J. Heather, K. Flower, S. Isaac and A. J. Sinclair

*Journal of General Virology* vol. **90**, part 6, pp. 1450–1454

**Supplementary Fig. S1.** Effect of methylation and Zta expression on the activity of the *erg1* promoter in U2OS cells. [PDF] (31 KB)

  
  
