# Supplementary material for: The Epstein–Barr virus lytic cycle activator Zta interacts with methylated ZRE in the promoter of host target gene egr1
Source: J Gen Virol. 2009 Jun;90(Pt 6):1450–4. doi: 10.1099/vir.0.007922-0 (PMC2885059; doi:10.1099/vir.0.007922-0)
Supplement: [Supplementary figure] [file supp_90_6_1450__1.pdf]

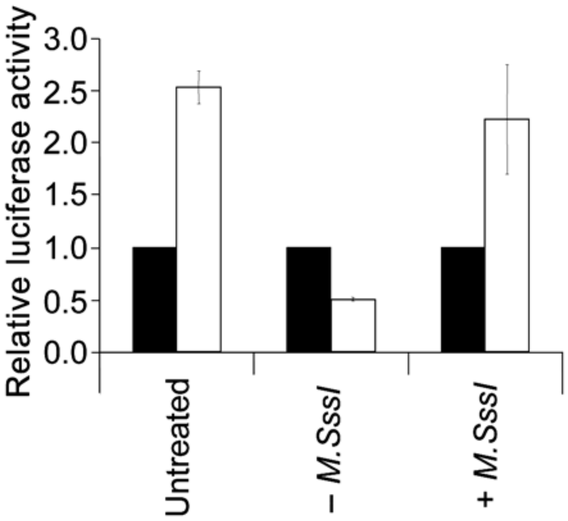

Supplementary Fig. S1. Effect of methylation and Zta expression on the activity of the *erg1* promoter in U2OS cells. USOS cell lines were transfected with the indicated plasmids using Effectene (Qiagen). After 48 h cell extracts were prepared and assayed for luciferase activity using the luciferase assay system (Promega). The data for each type of plasmid [luciferase activity (µg protein)$^{-1}$] are expressed relative to the activity levels seen in the absence of Zta, together with the SD, which was derived from at least two experiments. The reporter plasmid used was *egr1* (–504/+9)LUC (Chang *et al.*, 2006). The plasmid was transfected in an untreated form; or it underwent a mock methylation reaction (–*M.SssI*); or it went through an overnight methylation reaction with the methyl transferase *M.SssI* (New England Biolabs) (+*M.SssI*) followed by purification on a QIAprep column (Qiagen). Prior to transfection, the extent of methylation was evaluated by a diagnostic digestion with the methylation-sensitive restriction enzyme *Bst*UI. The open bars represent transfections undertaken with pBabe BZLF1 (Hicks *et al.*, 2003), while the filled bars represent transfections undertaken with the 'empty' pBabe vector.

Heather, J., Flower, K., Isaac, S. & Sinclair, A. J. (2009). The Epstein–Barr virus lytic cycle activator Zta interacts with methylated ZRE in the promoter of host target gene *egr1*. *J Gen Virol* **90**, 1450–1454.
